# RANKL inhibition reduces lesional cellularity and Gα_s_ variant expression and enables osteogenic maturation in fibrous dysplasia

**DOI:** 10.1038/s41413-023-00311-7

**Published:** 2024-02-20

**Authors:** Luis F. de Castro, Jarred M. Whitlock, Zachary Michel, Kristen Pan, Jocelyn Taylor, Vivian Szymczuk, Brendan Boyce, Daniel Martin, Vardit Kram, Rebeca Galisteo, Kamran Melikov, Leonid V. Chernomordik, Michael T. Collins, Alison M. Boyce

**Affiliations:** 1 Skeletal Disorders and Mineral Homeostasis Section, National Institute of Dental and Craniofacial Research, National Institutes of Health, Bethesda, MD USA; 2 Section on Membrane Biology, Eunice Kennedy Shriver National Institute of Child Health and Human Development, National Institutes of Health, Bethesda, MD USA; 3 Metabolic Bone Disorders Unit, National Institute of Dental and Craniofacial Research, National Institutes of Health, Bethesda, MD USA; 4 Department of Plastic and Reconstructive Surgery, The Johns Hopkins Medical Institutions, Baltimore, MD USA; 5 Department of Pathology and Laboratory Medicine, University of Rochester Medical Center, Rochester, NY USA; 6 NIDCR Genomics and Computational Biology Core, National Institute of Dental and Craniofacial Research, National Institutes of Health, Bethesda, MD USA

**Keywords:** Calcium and phosphate metabolic disorders, Bone, Metabolic bone disease

## Abstract

Fibrous dysplasia (FD) is a rare, disabling skeletal disease for which there are no established treatments. Growing evidence supports inhibiting the osteoclastogenic factor receptor activator of nuclear kappa-B ligand (RANKL) as a potential treatment strategy. In this study, we investigated the mechanisms underlying RANKL inhibition in FD tissue and its likely indirect effects on osteoprogenitors by evaluating human FD tissue pre- and post-treatment in a phase 2 clinical trial of denosumab (NCT03571191) and in murine in vivo and ex vivo preclinical models. Histological analysis of human and mouse tissue demonstrated increased osteogenic maturation, reduced cellularity, and reduced expression of the pathogenic Gα_s_ variant in FD lesions after RANKL inhibition. RNA sequencing of human and mouse tissue supported these findings. The interaction between osteoclasts and mutant osteoprogenitors was further assessed in an ex vivo lesion model, which indicated that the proliferation of abnormal FD osteoprogenitors was dependent on osteoclasts. The results from this study demonstrated that, in addition to its expected antiosteoclastic effect, denosumab reduces FD lesion activity by decreasing FD cell proliferation and increasing osteogenic maturation, leading to increased bone formation within lesions. These findings highlight the unappreciated role of cellular crosstalk between osteoclasts and preosteoblasts/osteoblasts as a driver of FD pathology and demonstrate a novel mechanism of action of denosumab in the treatment of bone disease.

TRIAL REGISTRATION: ClinicalTrials.gov NCT03571191

## Introduction

Fibrous dysplasia (FD) is a rare mosaic disorder that can lead to fracture, pain, and physical disability. Somatic gain-of-function Gα_s_ variants are acquired during embryogenesis, altering skeletal stem cell differentiation and leading to expansile lesions of highly proliferative, partially mineralized fibro-osseus tissue.^1^ In the craniofacial skeleton, FD lesion expansion causes facial asymmetry and functional deficits, such as vision and hearing loss.^2^ Lesions in weight-bearing bones can fracture and deform, leading to pain and ambulation impairment.^3^ There are no approved treatments, and there is a critical need for targeted therapies.

The development of effective treatments for FD has been hindered by knowledge gaps in the interplay between lesional cell populations. Abnormal osteogenic cells harboring pathogenic Gα_s_ variants constitute the dominant lineage and lead to signature histopathological features, including fibrosis, altered bone formation, and loss of hematopoietic/fatty marrow.^1^ Osteoclasts are prominent in FD tissue; however, their role in FD pathogenesis is unknown. Growing evidence suggests that targeting receptor activator of nuclear kappa-B ligand (RANKL), a cytokine that promotes osteoclastogenesis, is a promising potential treatment strategy.^4^ RANKL released by osteoprogenitors and other cell types binds to RANK on the surface of osteoclast precursors and elicits osteoclastogenesis, and these committed cells fuse and form mature, multinucleated active osteoclasts that seal the exposed mineralized matrix and form a resorption pit.^5^ Our group recently reported results from a phase 2 trial of denosumab, a RANKL monoclonal antibody, in adults with FD, demonstrating potent inhibitory effects on FD lesions as measured by circulating bone turnover markers and ^18^F-NaF in PET/CT lesional activity.^4^ This clinical improvement was associated with a reduction in the proliferation of FD osteoprogenitors and increased mineral formation within the lesions. Recent work in murine models also supports the notion that RANKL inhibition enhances lesional mineralization and limits its expansion, in part through attenuation of osteoclast resorption.^6,7^ Understanding the effects of RANKL inhibition on FD cellular behavior and lesion composition therefore has the potential to inform understanding of FD and its management and the interplay between osteoclastic and osteoblastic lineages in health and disease. Here, we characterized the effects of RANKL inhibition in human FD tissue and in preclinical models to define the cellular effects on FD osteoprogenitors.

## Results

### Denosumab decreased the expression of gain-of-function *GNAS* and cellularity in FD tissue

**Fig. 1:**
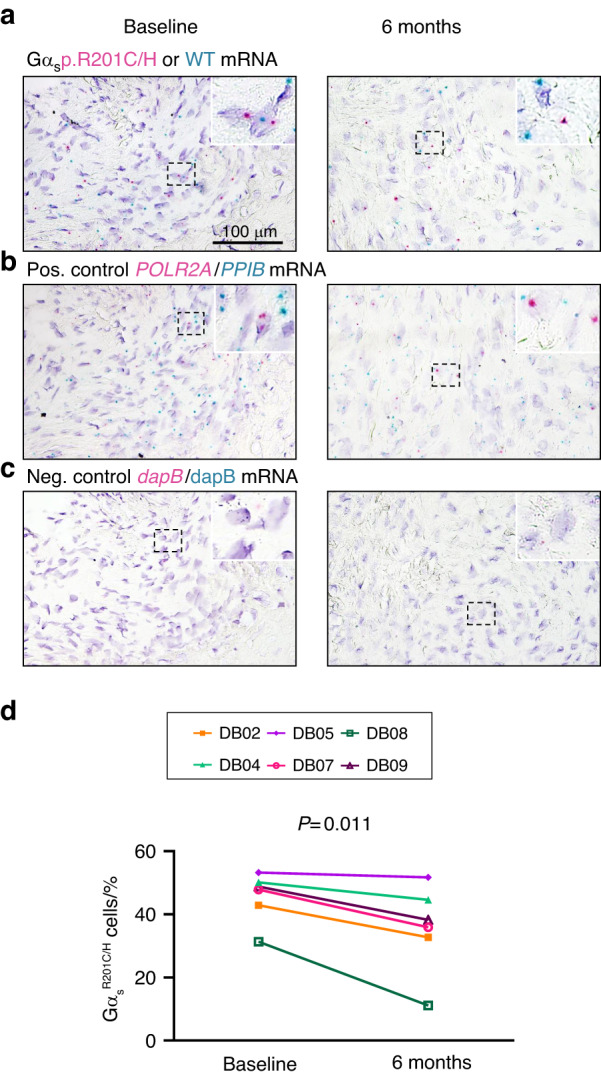
Denosumab treatment decreased Gα_s_^R201C/H^ mRNA expression in human FD tissue. **a** Representative images of Gα_s_^R201C^ (red dots, right image) and Gα_s_^R201H^ (red dots, left image) mRNA staining before and after denosumab treatment compared to that of WT Gα_s_. **b** Positive control mRNA staining of POLR2A (red dots, low expression) and PPIB (green dots, high expression) in the matching field of sections consecutive to those in **a**. **c** Negative control mRNA staining of the bacterial gene dapB in the matching field of sections consecutive to **a** and **b**. **d** Ratio of cells expressing Gα_s_^R201C/H^ mRNA with respect to cells expressing only WT Gα_s_ mRNA in each FD biopsy before and after denosumab treatment, calculated as the number of nuclei in the immediacy of red dots (Gα_s_^R201C/H^ mRNA) with or without the presence of green dots (WT Gα_s_ mRNA) divided by the number of nuclei in the immediate area of only green dots (WT Gα_s_ mRNA)

We assessed the abundance of Gα_s_^R201C/H^-expressing cells in FD tissue before and after treatment with denosumab via the novel technique Basescope™ using biopsies from a phase 2 clinical trial.^4^ First, in collaboration with ACD, we used cultured bone marrow stromal cells (BMSCs) from FD patients harboring the GNAS p.R201C and p.R201H variants and from healthy wild-type (WT) volunteers to select, optimize and validate probes specific for the WT Gα_s_, Gα_s_^R201C^ and Gα_s_^R201H^ mRNAs (Fig. S1). Then, we evaluated the expression of these variants in FD tissue before and after denosumab treatment using a dual-color duplex system in which WT Gα_s_ mRNA molecules are labeled with green dots and Gα_s_^R201C^ or Gα_s_^R201H^ mRNA molecules are labeled with red dots (Fig. 1a). As positive controls, we used mRNA probes to detect both weakly and abundantly expressed housekeeping genes (*POLR2A* and *PPIB,* respectively; Fig. 1b), and a probe detecting the bacterial gene *dapB* was used as a negative stain control (Fig. 1c). The number of cells expressing Gα_s_^R201C^ or Gα_s_^R201H^ mRNA in each section was counted as the number of nuclei within the immediate region of red dots divided by the number of cells expressing only WT Gα_s_ mRNA,- considered as nuclei within the immediate region of green dots but not red dots. Treatment with denosumab reduced the relative frequency of Gα_s_^R201C^ - or Gα_s_^R201H^-mRNA-expressing cells by 25% in all patients in the present study (Fig. 1d).

**Fig. 2:**
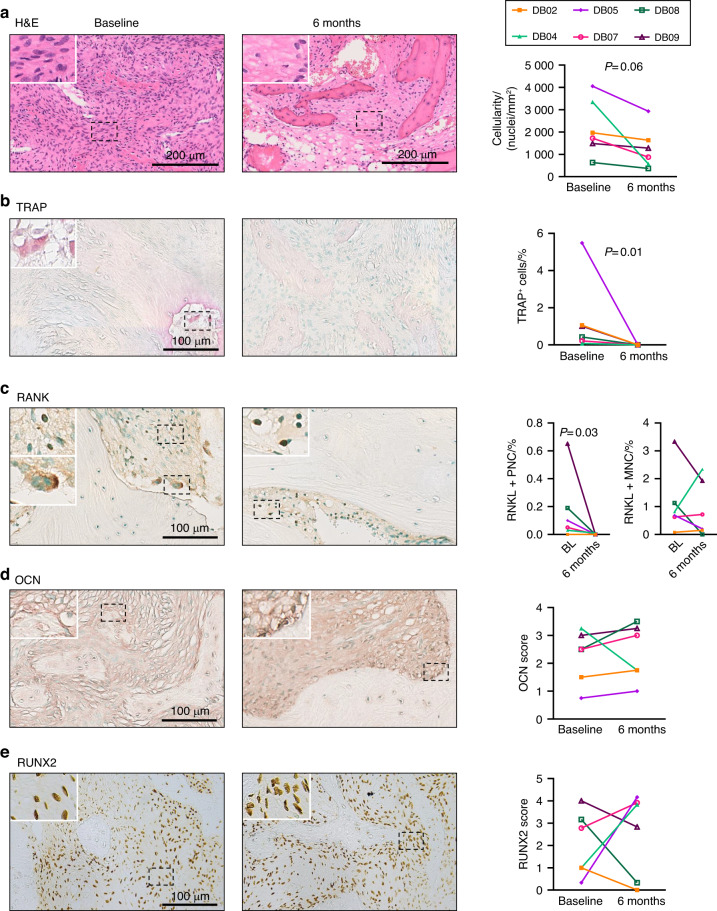
Histological changes in human FD post-denosumab treatment. **a** Representative images of changes in FD tissue histology before (baseline) and after treatment with denosumab (6 months). H&E staining from subject DB04 showing decreased cellularity. **b** Loss of TRAP activity after denosumab treatment (DB07). **c** Immunostaining and quantification of RANK. Clearance of RANK^+^ multinucleated cells was observed after denosumab treatment, while RANK^+^ mononuclear cells remained unchanged after treatment (DB09). **d** Five out of six biopsies demonstrated small, nonsignificant increases in osteocalcin staining. **e** There was no change in the expression level of RUNX2, which has broad baseline expression within the fibrotic tissue. **d** and **e** scores were evaluated by 3 independent readers. PNC Polynuclear cells, MNC mononuclear cells

FD tissue cellularity was assessed via H&E staining of human biopsies before and after denosumab treatment, and a lower FD cell density was observed in all patients after treatment (Fig. 2a), consistent with a previously reported decrease in proliferation markers.^4^ To evaluate the impact of osteoclast ablation, we assessed TRAP enzymatic activity and performed RANK immunodetection. As expected, both staining methods revealed the absence of multinucleated osteoclasts, while the number of RANK^+^ mononuclear osteoclast precursors remained unchanged (Fig. 2b, c). Although we previously reported increased expression of the osteocyte marker SOST upon administration of denosumab,^4^ we did not observe a consistent trend in the staining level of the early and late osteogenic markers RUNX2 and osteocalcin (OCN), although samples from 5/6 patients showed increased OCN staining (Fig. 2d, e).

### Anti-RANKL treatment in an inducible mouse model of fibrous dysplasia successfully mimicked the response of FD patients to denosumab

**Fig. 3:**
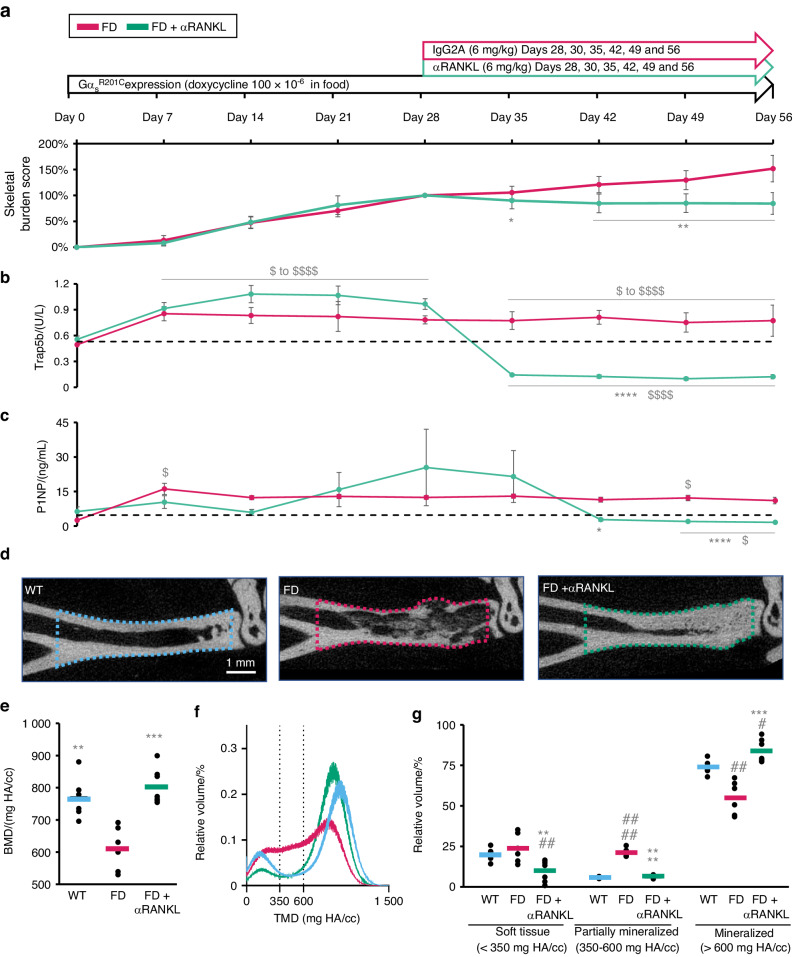
αRANKL treatment decreased the disease burden in mice with FD and increased lesional mineralization. **a**–**c** Longitudinal data showing the experimental scheme, longitudinal disease progression and effect of αRANKL therapy in mice with FD. **a** An X-ray-based skeletal disease burden tool was developed to assess disease activity in mice. The data were normalized to the individual disease burden score (100%) at the start of αRANKL/IgG2A control treatment and 28 days after FD lesion induction by doxycycline administration. Serum **b** Trap5b and **c** P1NP levels, which are markers of bone resorption and formation, respectively, were assessed at the indicated time points. **d** Representative μCT mid-sagittal plane images of standardized regions of interest of the distal tibia analyzed in **e**–**g**. **e** Average bone mineral density (BMD) values of the regions of interest. **f** Individual voxel density distributions across the regions of interest of group averages (WT is blue, FD is magenta and FD + αRANKL is green). **g** Relative volumes of soft, partially mineralized, and mineralized tissue within the regions of interest as defined by the cutoff values in **f**. * is *P* < 0.05, ** is *P* < 0.01, *** is *P* < 0.001 and **** is *P* < 0.000 1 vs. the untreated FD group. $ is *P* < 0.05, $$$$ is *P* < 0.000 1 vs. baseline. # indicates *P* < 0.05, ## indicates *P* < 0.01 and #### indicates *P* < 0.000 1 vs. the WT group

To overcome the replicability limitations of the study of human biopsies and expand our observations of RANKL inhibition in FD, we modeled the treatment course using an inducible FD mouse model in which the human transgene Gα_s_^R201C^ is expressed in the appendicular skeleton while the mice are given doxycycline. This model has proven to show highly reproducible mosaic-like and site-specific FD lesions^6,8^ (Fig. S3). Lesion progression was evaluated in these mice with an in vivo X-ray-based disease burden scoring system that was able to faithfully capture disease progression (Table S1, Fig. S2a). Four weeks after FD induction, the mice developed lesions in their appendicular skeleton, and αRANKL or isotype control antibodies were administered for four additional weeks. Mice were euthanized on Day 56, two days after the last injection (Fig. 3a). Disease progression analysis revealed a reduction in the αRANKL-treated mice compared to the IgG2A-treated controls (Fig. 3a). The bone turnover markers Trap5b and P1NP increased above baseline levels one week after disease induction and decreased after αRANKL treatment, remaining lower than baseline levels from weeks one to three after treatment started (Fig. 3b, c).

As FD lesions progress in mice, bone is first resorbed (Fig. S3c, d) and then gradually replaced by low-density fibro-osseous tissue, which also invades the soft tissue marrow space (Fig. S3e, f, middle panel in Fig. 3d), leading to an overall decrease in bone mineral density (Fig. 3e). To investigate this process in more detail, we plotted the µCT voxel density distribution of FD tissue (Fig. 3f) within a volume of interest (Fig. 3d, dotted lines). In these distributions, we identified tree shoulders corresponding to soft tissue (<350 mg HA/cc), partially mineralized tissue (350–600 mg HA/cc) and mineralized tissue (>600 mg HA/cc) and calculated their relative volumes within the volume of interest (Fig. S4). Untreated FD tissue showed no changes in soft tissue content but developed an almost 4-fold increase in partially mineralized tissue, with a corresponding decrease in mineralized tissue (Fig. 3f, g). In lesions from animals treated with αRANKL, these changes were reversed, as the average BMD and content of partially mineralized tissue were similar to those of normal bone. Interestingly, the soft and ossified tissue contents were lower and greater, respectively, in these mice than in WT mice, revealing an overall increase in bone content at the expense of the intramedullary marrow space (observable in Fig. 3d, left image).

### Mouse FD histology supports and expands the observations in human biopsies

**Fig. 4:**
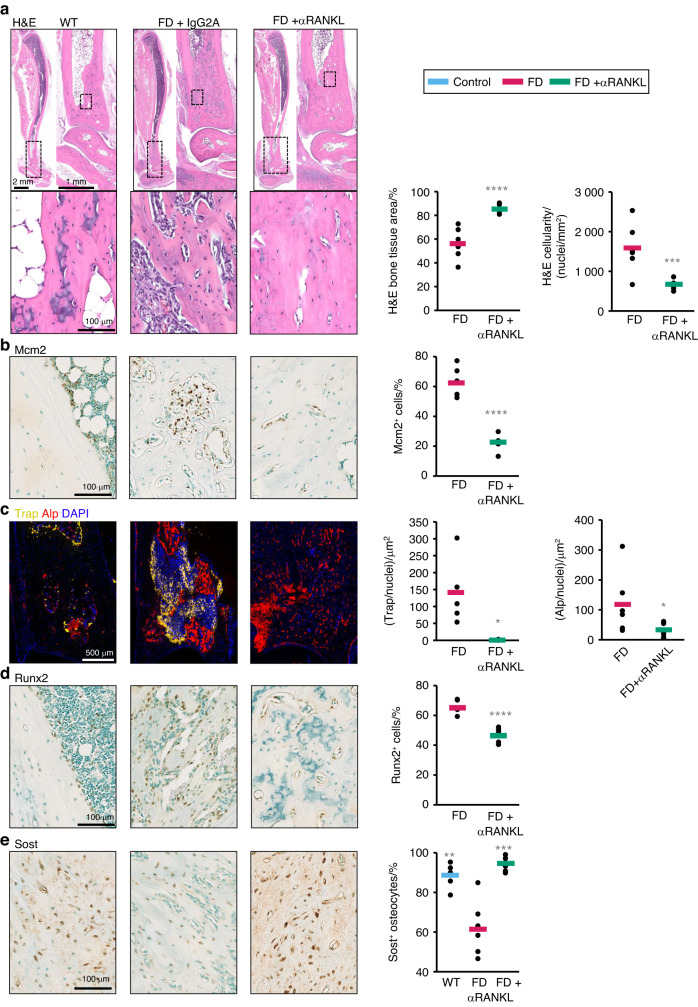
αRANKL decreased proliferation and increased differentiation in murine FD lesions. **a** H&E staining of the distal tibiae of WT mice and mice with FD and analysis of the cellularity and bone content (mineralized tissue area) of the lesioned areas with or without αRANKL treatment. **b** MCM2 immunostaining of distal tibiae from WT mice as well as treated and untreated mice with FD. **c** TRAP, ALP enzymatic activity and DAPI nuclear staining; **d** RUNX2 immunostaining of the distal tibiae of WT mice, and treated and untreated mice with FD and **e** SOST immunostaining of cortical bone in the distal tibiae of WT as well as treated and untreated FD mice. The cortical bone in the distal tibiae of WT mice was used to calculate the physiological level of SOST positivity within osteocytes. The data are shown as individual mice (dots) and group averages (bars). * is *P* < 0.05, ** is *P* < 0.01, *** is *P* < 0.001 and **** is *P* < 0.000 1 vs. the FD group

The availability of murine FD histology allowed us to further investigate trends observed in human biopsies. Consistent with the findings of previous reports in human tissue,^4^ analysis of demineralized H&E sections of murine FD lesions revealed increased bone matrix content and decreased cellularity as a result of RANKL inhibition (Fig. 4a), which was associated with a decreased prevalence of Mcm2^+^ proliferating fibroblasts^9^ (Fig. 4b). As observed in human FD samples, lesional TRAP^+^ osteoclasts were completely abrogated (Fig. 4c). Whereas in human samples, the levels of RUNX2 positivity within FD lesions were highly variable before and after denosumab treatment, mouse studies have helped clarify the differentiation status of BMSCs in FD lesions, revealing a significant decrease in RUNX2-expressing FD cells in αRANKL-treated mice (Fig. 4d), together with a reduction in the early to mid-osteogenic differentiation marker ALP (Fig. 4c). Confirming our observations in human biopsies,^4^ a dramatic increase in the number of osteocytes positive for SOST was observed in response to αRANKL treatment (Fig. 4e).

### RNAseq analysis revealed an FD genetic signature that was reversed by anti-RANKL therapy

**Fig. 5:**
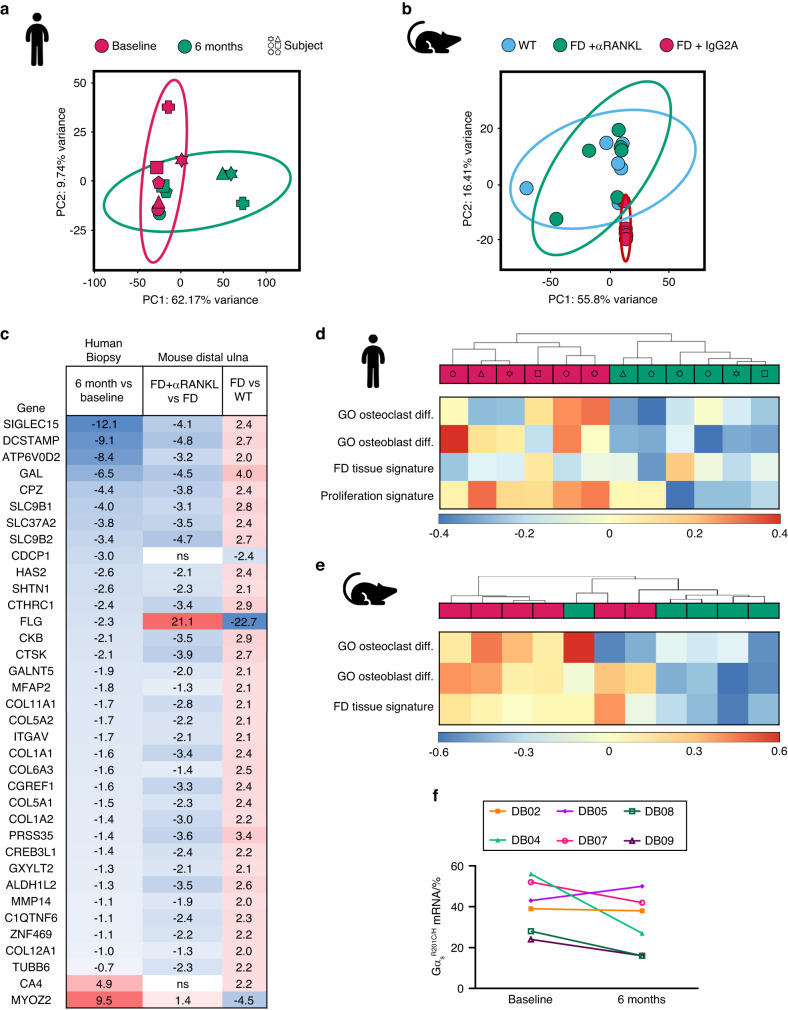
RANKL inhibition reverted the FD transcriptomic signature in human and mouse tissues. **a** RNA-seq principal component analysis (PCA) showing segregation of the transcriptomic profiles of human FD tissue before and after denosumab treatment. **b** PCA of mouse FD tissue showing segregation of the αRANKL- and IgG-treated FD tissue group and overlap with site-matched WT tissue. **c** FD signature genes significantly regulated by denosumab in human FD tissue (left column). **d** Unsupervised clustering of mRNA transcriptome gene set variation analysis (GSVA) scores against selected molecular signatures and gene sets in human samples. **e** Unsupervised clustering of whole-transcriptome GSVA scores against selected molecular signatures and gene sets in mouse samples. **f** Relative abundance of mutant *GNAS* reads in human RNAseq data, showing a decreased abundance of mutant Gα_s_ in 4/6 samples. “Diff.” means “differentiation”

To identify novel genes involved in FD pathogenesis and the effects of αRANKL therapy, we performed mRNA sequencing of human and mouse FD tissues. Although the transcriptomic principal component analysis (PCA) of human FD tissue showed limited segregation between baseline and post-denosumab biopsies in the PCA space, as shown by the 95% confidence intervals (CIs), significant changes in 1 296 genes were found (Fig. 5a, Table S3). Transcripts from αRANKL-treated murine FD tissue were better segregated from control FD tissue receiving the isotype IgG2A antibody and overlapped with those from unaffected murine bone, indicating a normalizing effect on the bone tissue expression profiles (Fig. 5b). This change reflected a significant difference in the expression of 3 324 genes between FD control tissue and site-matched unaffected tissue and 2 596 genes between αRANKL-treated FD tissue and FD control tissue but only 65 genes between αRANKL-treated FD tissue and unaffected tissue (Tables S4–S6). Sequencing datasets are available at the NCBI GEO repository under series accession number GSE250357.

By filtering the 276 (out of the 3 324) genes with ±4-fold differential expression in murine FD tissue, we were able to define an FD tissue transcriptomic signature. Of these, 202 genes had an identified human ortholog (Tables S7 and S8). Of the genes identified in this FD genetic signature, 36 were significantly modulated in human FD biopsies following denosumab treatment (Fig. 5c). The expression of most of these genes was opposite to that observed in the comparison between IgG-treated FD and wild-type mice. Changes in 34/36 of these genes were detected in αRANKL-treated mice, which exhibited similar expression patterns.

FD transcriptomes were investigated using the publicly available “Gene Ontology: Biological Process” genetic expression dataset. Unsupervised clustering by gene set variation analysis (GSVA) demonstrated strong segregation of human and mouse samples according to treatment status and strong associations with signatures related to osteoblast and osteoclast differentiation (Fig. 5d, e). Similarly, a number of genes related to osteoclast differentiation and activity were strongly downregulated upon RANKL inhibition in mice and human FD (Fig. S5a, b). Further analysis of representative osteogenic differentiation markers revealed negative changes in early/mid-differentiation markers (*RUNX2, SP7*, and *ALPL*) but strong increases in *SOST* and *BGLAP* (osteocalcin) in human samples and *Sost* in mouse samples, supporting the increase in the amount and maturation of FD lesional bone observed after αRANKL treatment (Fig. S5c, d). In addition, we analyzed the expression of well-known osteoclast-osteoprogenitor crosstalk genes in these datasets, most of which were upregulated in FD tissue compared to WT bone (Table S4). The expression of most of the genes encoding factors released by osteoprogenitors and that act on osteoclast progenitors did not change after αRANKL treatment, except for *SEMA3A* and *TNFSF11* (which encode RANKL) in human tissue, which were significantly decreased by denosumab administration (Fig. S5e, f). On the other hand, the expression of most genes associated with osteoclast-to-osteoprogenitor signaling dramatically decreased with αRANKL treatment (Fig. S5g, h).

The human transcriptome demonstrated strong segregation with the Meta-PCNA tissue proliferation signature, which included 132 genes coexpressed with PCNA in healthy tissues from 27 solid organs^10^ (Fig. S5e), further supporting the effect of RANKL inhibition on FD lesion cellularity and proliferation. The expression of pathogenic Gα_s_ variants was detected with sufficient sensitivity in all human specimens (374-3 055 reads) and decreased in 4/6 post-treatment samples (Fig. 5f), supporting our observation using variant-specific probes, although this overall decrease of approximately 20% in pathogenic Gα_s_ allele frequency did not reach statistical significance (*P* = 0.15). We also observed a significant decrease in the expression of the transgene Gα_s_^R201C^ in mice with FD treated with αRANKL (Table S5); however, this observation cannot be confidently used to support the decreased Gα_s_ variant expression in humans, as Gα_s_^R201C^ in these mice is not expressed under its endogenous regulation.

### ex vivo FD cellular model showed that FD osteoprogenitors depend on osteoclasts for proliferation

**Fig. 6:**
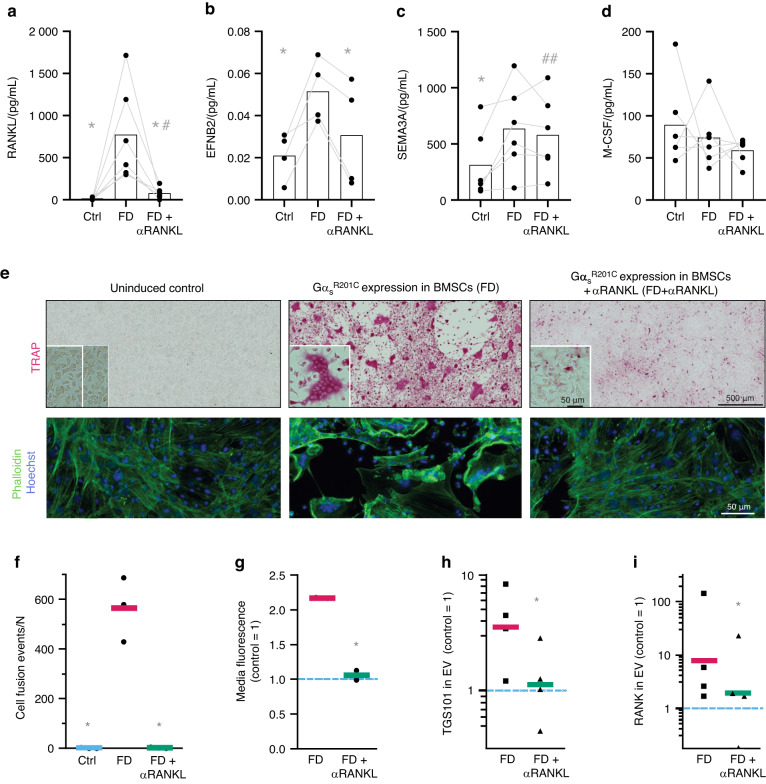
An ex vivo FD cellular model was used to confirm the increased osteoclast formation and the anti-osteoclastic effect of RANKL inhibition in FD lesions. **a**–**d** Four days after the administration of doxycycline, increased levels of the osteoclastogenic factors RANKL, EFNB2 and SEMA3A were detected in 24-h conditioned media, although no changes were observed in M-CSF. In cultures treated with αRANKL levels of RANKL and EFNB2 were comparable to control (uninduced) cultures. **e** Bone marrow cultures develop large TRAP^+^ osteoclasts after the addition of doxycycline, which are absent when αRANKL is administered. **f** Osteoclast fusion efficiency assay showing no significant cell fusion events in uninduced (Ctrl) or αRANKL-treated cultures. **g** In vitro bone resorption assay showing the detection of resorption degradation products in the media of induced cultures in comparison to uninduced cultures (blue dashed line). αRANKL administration normalized these levels. **h**, **i** Extracellular vesicles (EVs) were isolated from 24h conditioned media and lysed for protein extraction, identification and quantification by Western blotting. RANK (the RANKL receptor) and the EV marker TGS101 were detected. **h** Densitometric quantification of TGS101 expression in EVs from uninduced control cultures (blue dashed line) and FD cultures with or without αRANKL treatment via Western blotting (not shown). **e** Densitometric quantification of Western blot (not shown) data for RANK in EVs from uninduced control cultures (blue dashed line) and FD cultures with or without αRANKL treatment. *P < 0.05 vs. FD; #*P* < 0.05, ##*P* < 0.01 vs. the control

To further evaluate the contribution of osteoclastogenesis to the abnormal behavior of FD osteoprogenitors, we utilized an ex vivo model of FD to evaluate the mechanism of osteoclast to osteoprogenitor coordination.^11^ This ex vivo model was established using whole bone marrow from uninduced FD mice and contained adherent cells of the monocytic lineage and BMSCs, progenitors of osteoclasts and osteoblasts/osteocytes respectively. The addition of doxycycline to the culture media induced Gα_s_^R201C^ expression only in BMSCs (Fig. S6). After induction, BMSCs released osteoclastogenic factors such as RANKL, EFNB2 and SEMA3A (Fig. 6a-c). Interestingly, M-CSF production did not change upon Gα_s_^R201C^ expression (Fig. 6d), and FAS-L was undetectable under all culture conditions (data not shown; ELISA detection range, 31.2–2 000 pg/mL). These factors induced the fusion of mononuclear precursors into TRAP^+^ multinucleated osteoclasts (Fig. 6e, f), which can resorb hydroxyapatite in vitro (Fig. 6g).

**Fig. 7:**
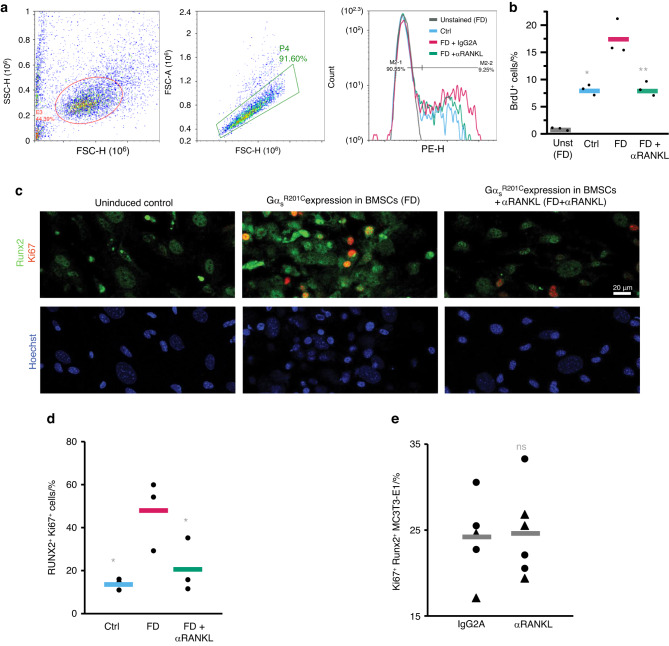
αRANKL abrogated the increase in BMSC proliferation in ex vivo bone marrow cultures after Gα_s_^R201C^ expression. **a** Representative gating protocol for evaluating the number of cultured cells that incorporated BrdU, demonstrating DNA replication. First, the cells were analyzed for side scatter (SSC) and forward scatter (FSC) heights to identify single cells. Next, cells from the first gate (E3) were analyzed for FSC area vs. height to distinguish single cells from doublets. Finally, ~5 000 cells per group were evaluated for BrdU incorporation. **b** Evaluation of the percentage of cells that underwent BrdU incorporation. **c** Ki67 (red), Runx2 (green) and nuclear Hoechst staining (blue) of ex vivo bone marrow cultures indicating an increase in the proliferation and abundance of Runx2^+^ BMSCs after Gα_s_^R201C^ expression normalized upon αRANKL administration. **d** Relative abundance of RUNX2^+^ Ki67^+^ cells in each group. **e** Administration of αRANKL did not change the abundance of Ki67^+^ cells among the Runx2^+^ cells in cultures of MC3T3-E1 clone 4 (triangles) or 14 (dots) preosteoblasts. The data are shown as individual cultures (dots) and group averages (bars). *P < 0.05, **P < 0.01 vs. the FD group

RANK-containing extracellular vesicles (EVs) have been proposed to constitute a major mechanism by which osteoclasts control osteoprogenitor proliferation and differentiation to modulate bone formation after bone resorption in a process known as remodeling coupling.^12–14^ In our study, following the formation of osteoclasts, abundant EVs were readily detected in the media, as confirmed by the expression of the EV marker TGS101 (Fig. 6h). These EVs contained RANK, indicating an increase in this osteoclast-osteoprogenitor EV-mediated coupling mechanism upon FD induction (Fig. 6i). Under these conditions, an increase in the DNA content of single cells from these cultures was observed, as measured by BrdU incorporation, supporting the increase in BMSC proliferation observed in human and murine FD tissue (Fig. 7a, b). Moreover, the increase in BrdU incorporation was completely blocked by RANKL inhibition. To assess whether the increase in cell proliferation observed was due to elevated BMSC/osteoprogenitor proliferation, we assessed our cultures via immunofluorescence microscopy. We found that Runx2^+^ osteoprogenitors exhibited elevated Ki67^+^ expression (a marker of cell proliferation) and a gross increase in cellularity (Fig. 7c, d). RANKL inhibition via αRANKL in FD cultures normalized the increase in the levels of some of the osteoprogenitor-released osteoclastogenic factors in the media (Fig. 6a–c), and multinucleated osteoclasts were no longer observable or active (Fig. 6e-g). RANK-containing EVs were decreased (Fig. 6h, i), and the doxycycline-induced osteoprogenitors no longer exhibited increased proliferation (Fig. 7c, d), consistent with our previous observations in human cultured FD BMSCs treated with denosumab.^15^ However, when αRANKL was added to cultures of MC3T3-E1 osteoprogenitors, no effect on cell proliferation was observed (Fig. 7e), Taken together, these findings indicate that osteoclasts contribute to the proliferation of abnormal FD osteoprogenitors, possibly through the release of TGS101^+^ RANK^+^ EVs, which were previously associated with osteoclast-osteoblast coupling,^6,8,16^ among other factors.

## Discussion

In this study, RANKL inhibition induced marked effects on FD osteoprogenitors beyond its expected antiresorptive action, demonstrating the importance of osteoclast activation and perturbed coupling as key drivers of FD pathogenesis. These results support a model in which inhibition of RANKL-mediated osteoclastogenesis promotes lesional bone formation by inhibiting the proliferation of FD osteoprogenitors, thus enabling their maturation. These alterations likely represent clinically relevant changes because FD cell proliferation is associated with increased lesional activity and expansion,^15,17,18^ and mineralization defects are associated with bone weakness, deformities, and fractures.^19,20^ Taken together, the findings from this study support the targeting of osteoclasts through RANKL inhibition as a mechanistically driven treatment strategy.

For the first time, we report a reduction in gain-of-function Gα_s_ variant expression in human tissue after denosumab treatment using a novel variant-specific mRNA staining technique using probes we previously validated in human FD BMSC cultures. Tissue RNAseq analyses supported these findings, revealing a 20% average decrease in the frequency of mutant alleles, although the difference was not statistically significant. However, these RNA-based techniques cannot discern whether the detected decrease in Gα_s_ variant expression in treated patients was due to an antiproliferative effect that more robustly impacted mutant cells than WT cells within FD tissue or to a drug-induced decrease in the expression of mutant versus wild-type Gα_s_ variants by mutation-bearing cells. We also observed a decrease in the expression of this transgene in our mice after treatment with αRANKL (Table S5); however, because Gα_s_ expression is controlled by a tetracycline-inducible promoter, this is not an ideal model for investigating its potential regulation by RANKL. Taken together, our findings suggest that the antiproliferative effects of RANKL inhibition may preferentially impact Gα_s_ variant-expressing osteoprogenitors compared with wild-type cells. Interestingly, a decrease in the proportion of variant-bearing cells has been proposed as a potential mechanism for the established age-related reduction in FD lesion activity, which is associated with reduced skeletal complications in adulthood.^18,21,22^ This finding suggested that targeting Gα_s_ variant expression is a potential therapeutic strategy for FD and may contribute to the clinical improvements observed with denosumab treatment.^4,23^ However, further studies are needed to confirm these findings and to determine the mechanisms by which denosumab affects Gα_s_ expression.

FD histopathological and RNAseq analyses in both humans and mice demonstrated a broad pattern of αRANKL-driven osteogenic maturation. Both human and mouse FD tissue samples demonstrated high expression of early-to-mid-osteogenic differentiation markers, including RUNX2 and ALP; however, only murine tissue samples exhibited decreased RUNX2 and ALP staining upon RANKL inhibition. Interestingly, the mRNA level of the mid-stage differentiation marker osteocalcin was decreased in mouse FD amples but increased in human FD samples, and in both cases, the late-stage differentiation marker sclerostin was dramatically upregulated after RANKL inhibition. Taken together, the findings from both human and mouse tissues demonstrated that RANKL inhibition results in consistent movement toward a more differentiated state. The discrepancies in the expression of early-to-mid-osteogenic maturation markers between species likely reflect the overall greater bone content in mouse versus human FD tissue (Fig. 2a vs. 4a). This greater mineralization status observed in mice may reflect an inherent, species-dependent limitation of this model. The findings may also be confounded by size effects: while a complete long bone section was analyzed in mice, a very small biopsy in relation to the complete bone was analyzed in humans.

A comparison of mouse FD and wild-type tissue provided the opportunity to generate an FD transcriptional signature composed of 202 strongly modulated genes that had human orthologs. Of these, 36 demonstrated significantly altered expression after denosumab treatment in humans. Most changes were opposite to those observed in the comparison of FD vs. wild-type bone in mice, suggesting that denosumab likely induces a more “normalized” genetic expression profile in human FD tissue. The changes in the αRANKL-treated mice were strikingly similar, with 34/36 of these genes exhibiting similar expression patterns. These findings support the robustness of the murine model for identifying RANKL inhibitory effects in humans and the potential utility of this genetic signature for further exploring the biology of FD in future studies.

When we assessed the associations of osteoprogenitors-osteoclast crosstalk factors in our models, we observed that, in addition to RANKL (the Tnfsf11 gene), some additional osteoclastogenic factors were mildly increased in FD compared to WT cells or tissue (Table S4, Fig. 6a–d). αRANKL therapy normalized the levels of a subset of the genes. Interestingly, the expression of M-CSF, an essential factor for osteoclastogenesis, was only moderately increased in murine FD tissue (Csf1 gene; Table S4) and not in FD tissue culture; moreover, M-CSF expression was not affected by αRANKL therapy. Among the pro-osteogenic factors produced by osteoclasts (also known as bone remodeling coupling factors), *Dpp4* was strongly upregulated in mouse FD tissue compared to that of WT bone (Table S4), and most of these factors were dramatically decreased after αRANKL treatment. Interestingly, in contrast to those in human FD tissue, *Dpp4* levels in mouse FD tissue increased after treatment with αRANKL. In addition, while RANK/RANKL signaling during bone remodeling has been broadly characterized as a pro-osteoclastogenic pathway, growing evidence suggests that signaling may actually have a bidirectional role in which vesicular RANK secreted from osteoclasts may promote osteoblast differentiation and bone formation through interaction with membrane-anchored RANKL on preosteoblasts.^24^ Using an ex vivo FD lesion model, we demonstrated that osteoclast formation was accompanied by the release of RANK-containing extracellular vesicles and increased osteoprogenitor cell proliferation, changes that were inhibited by αRANKL treatment. Taken together, these findings identify osteoclast-osteoblast lineage crosstalk as an essential mechanism for FD pathogenesis that can be targeted by RANKL inhibition. Additional studies building upon this work may provide further opportunities to capitalize upon these coupling pathways and identify novel therapeutic targets in FD.

Denosumab efficacy is transient, and discontinuation of treatment is associated with rapid loss of therapeutic efficacy, which resulted in severe increased bone turnover and hypercalcemia in one subject in our clinical trial.^4^ However, additional studies are needed to further understand and develop mitigating strategies for this rebound phenomenon in FD patients, including the potential utility of preclinical models. The findings from our current study offer insights that may inform the development of future mitigating strategies. Bisphosphonates are antiresorptive medications that incorporate into hydroxyapatite and lead to long-term inhibition of osteoclastogenesis; however, previous studies have shown that bisphosphonates do not improve FD lesion activity or composition.^18,25,26^ This phenomenon may result from the paucity of mineralized tissue within FD lesions, and it is possible that the increased mineralization generated by denosumab may subsequently enable lesional incorporation of bisphosphonates. If this is the case, bisphosphonate use prior to denosumab withdrawal, while new bone is forming, may offer a therapeutic advantage by providing sustained antiresorptive effects. Bisphosphonates have shown partial efficacy in mitigating post-denosumab rebound in other disorders^27^; however, in FD, they potentially offer the additional advantage of preserving denosumab-induced lesional mineralization. Further studies are needed to investigate the safety and efficacy of potential cotreatment regimens, as well as optimal dosing strategies for FD.

This study builds upon two previous reports exploring the effects of RANKL inhibition on murine FD models, reporting increased lesion mineralization and density.^6,7^ Liu Z et al. also observed decreased ALP expression and increased OCN^+^ mature osteoblasts,^6^ providing the first evidence that RANKL inhibition may impact osteogenesis. Our findings expand upon these findings and include novel investigations in denosumab-treated human tissue, which enabled the first detailed analyses of osteogenic cell differentiation and Gα_s_ variant expression patterns. Further research is needed to determine the implications of the timing of RANKL inhibition on disease development. Using a constitutive rGα_s_^R201C^ model, Palmisano et al. demonstrated that RANKL inhibition in young mice prevented the development of additional lesions.^7^ A phase 2 trial in children investigating the effect of denosumab on FD lesion progression is ongoing (NCT05419050).

The strengths of this study include the following: (a) This is the first report of in situ detection of pathogenic and wild-type Gα_s_ variants in FD and, to our knowledge, in any mosaic disease. This novel technique is invaluable for understanding the cellular dynamics and microenvironment of cancer and other mosaic diseases. (b) Systematic collection of pre- and post-treatment bone specimens as part of a prospective clinical trial provided a unique opportunity for detailed histopathological and molecular characterization of human tissue, a critical step in defining the role of RANKL and its inhibition in FD pathogenesis and treatment. (c) Investigations were integrated across patient specimens and model systems, allowing detailed inquiry into the inhibitory effects of RANKL on clinically relevant cellular processes. The *Prrx-1* inducible mouse model notably replicates key structural and histopathological features of human FD, and the ex vivo FD lesion model uniquely interrogates osteoclast–osteoblast coupling pathways. Decreased proliferation and increased osteogenic maturation in FD osteoprogenitors were thus demonstrated via multiple lines of inquiry, establishing a novel mechanism for targeting RANKL inhibition in FD. (d) mRNAseq analyses revealed the first comprehensive signature of genes significantly regulated in FD tissue. Comparison of mouse and human tissues verified this signature and revealed a robust list of genes normalized by αRANKL therapy, which may help identify new targetable pathways in FD. (e) This study involved the development of imaging assessments for murine studies, including an in vivo disease burden scoring system that shares similarities with the validated clinical method widely used in patients^28^ and advanced postmortem µCT analyses to detect differentially mineralized skeletal tissue. These sensitive tools will support future interventional studies in mice with FD.

This study also needs to be considered in the context of its limitations, which include the following: (a) Despite recruitment efforts, all subjects in the phase 2 clinical trial were women, and most had MAS-associated ovarian involvement. There is no pathophysiologic reason to expect sex-based differences in response to denosumab in patients with FD; however, it is possible that hormonal differences may have influenced bone turnover and gene expression patterns. Equal numbers of male and female mice were used in all groups without evidence of sex-based response differences, supporting the notion that the clinical study was likely not confounded by sex hormone effects. However, future clinical studies should prioritize the inclusion of diverse phenotypes, particularly men. (b) Analyses are subject to the inherent limitations of preclinical models. While the *Prrx-1* inducible model replicates key features of human FD,^8,29^ there are subtle but relevant differences, including a higher baseline proportion of bone content within fibro-osseous lesions and the inability to use this transgenic model to investigate the effects of RANKL inhibition on the regulation of FD Gα_s_ expression. (c) Bulk tissue for RNAseq can contain traces of unaffected tissue. It is possible that traces of neighboring hematopoietic marrow, which is highly proliferative, could have contributed to the lack of a detectable effect of RANKL inhibition on proliferation-related gene signatures in mice, despite histopathologic analyses, which conversely showed robust effects on cellularity and MCM2 expression in areas of FD.

In conclusion, this study demonstrated that in addition to its expected antiresorptive effects, denosumab reduces FD lesion activity by reducing the expression of pathogenic Gα_s_ variants in FD, decreasing FD cell proliferation and increasing osteogenic maturation, leading to increased lesional bone formation. These findings provide a new understanding of FD pathogenesis driven by unbalanced crosstalk between osteoclasts and preosteoblasts/osteoblasts and support the use of denosumab as a mechanistically driven treatment strategy.

## Materials and Methods

### Clinical trial design and oversight

A phase 2 open-label study was conducted at the NIH (NCT03571191). This investigator-sponsored study was supported by Amgen, Inc.; the study design, conduct, and analyses were performed by the investigators. The subjects received denosumab for 6 months at a dose of 120 mg every 4 weeks, with loading doses administered on weeks 2 and 3.^30^ Percutaneous FD lesion biopsies were performed at baseline and at 6 months in 6 of the 8 total adult subjects (Supplementary Fig. 1). Biopsies were deferred in 2 subjects: 1 due to the presence of isolated craniofacial FD and 1 due to intercurrent illness unrelated to the study intervention. Biopsy sites were chosen jointly by the investigators and surgeons to ensure that the procedure was minimally invasive, and biopsies were performed in the interventional radiology suite at the NIH Clinical Center using core needles under CT guidance. The same sites were biopsied before and after denosumab treatment.

### In vivo and ex vivo models of fibrous dysplasia

Mice expressing the Gα_s_^R201C^ variant were generated as previously described.^8^ At 10 weeks of age, Gα_s_^R201C^ expression was induced in the limbs by switching to doxycycline-supplemented food (100 × 10^−^^6^ Purina Mod LabDiet 5001; PMI Nutrition International, Saint Louis, MO). Six mice received an αRANKL antibody (6 mg/kg, BE0191, Bioxcell Lebanon, NH). Six Gα_s_^R201C^ mice as well as 6 littermate controls not harboring tet-GsαR201C received a rat IgG2A isotype control (6 mg/kg, BE0089, Bioxcell). All mice received subcutaneous injections on Days 28, 30, 35, 42, 49 and 56 after induction. Plasma was collected weekly, and on Day 58, the mice were euthanized, and tissue was collected. FD or control tissue was extracted from the distal ulna and radius, cleaned from the muscle and tendons, and snap-frozen for RNA analysis. Mice were then perfused with PBS and Z-fix fixative, and both hindlimbs were extracted for histology and µCT analysis.

Gα_s_^R201C^ expression was induced in ex vivo bone marrow cultures,^11^ which were subsequently treated with an αRANKL antibody. MC3T3-E1 clone 4 and 14 cells were cultured as previously described.^31^

### Mouse plasma measurements

Mouse blood (120 µL) was collected weekly via retro-orbital eye collection. At the time of euthanasia, 500–1 000 µL was obtained from the vena cava. Blood was stored in heparinized vials, and plasma was obtained. TRAP5b, CT-X and P1NP were measured using IDS ELISA kits SB-TR103, AC-06F1 and AC-33F1, respectively.

### Mouse X-rays and microCT

Mice were anesthetized with 2%–5% isoflurane, and X-ray images of the hind limbs were obtained on a Faxitron Ultrafocus system (Hologic, Marlborough, MA). A semiquantitative score was developed and validated to quantify the disease burden (Table S1). Three examiners independently evaluated the X-rays of both hindlimbs of each mouse at each timepoint.

The right hindlimbs were dissected and scanned using a Scanco µCT 50 at 10 µm, 70 kVp, 80 µA, and 900 ms integration time (Scanco, Wangen-Brüttisellen, Switzerland). The reconstructed images were analyzed with Analyze 14 (AnalyzeDirect, Overland Park KS) and calibrated against hydroxyapatite phantoms of known density. The volume of interest (VOI) was defined as the distal tibia sector between 500 µm below the fibula insertion point and 200 µm above the intermedium. The SmartTrace tool was used to outline the distal tibia every 50 µm in the sagittal direction, and then, the Propagate Objects tool was used to connect the 2D tracings, semiautomatically segmenting the VOI.

Following segmentation, the average bone mineral density (BMD) in milligrams of hydroxyapatite per cubic centimeter (mg HA/cm^3^) was obtained for the entire VOI. The distal tibiae were further analyzed by obtaining the number of voxels per BMD unit and binned into three categories: soft tissue (<350 mg HA/cm^3^), partially mineralized tissue (350-600 mg HA/cm^3^), and mineralized tissue (>600 mg HA/cm^3^).

### RNA extraction and sequencing

Mouse and human samples were snap-frozen, pulverized using an automated dry pulverizer (CP02 cryoPREP, Covaris, Woburn MA) and immediately transferred to TRIzol (Thermo Fisher Scientific). Phenol‒chloroform RNA extraction was carried out. cDNA synthesis, library construction and sequencing were performed by Novogene (University of California, Davis, CA). Briefly, messenger RNA was purified from total RNA using poly-T oligo-attached magnetic beads. After fragmentation, first-strand cDNA was synthesized using random hexamer primers, followed by second-strand cDNA synthesis, end repair, A-tailing, adapter ligation, size selection, amplification, and purification. The library was assessed with a Qubit fluorometer and real-time PCR for quantification and a bioanalyzer for size distribution detection. The quantified libraries were pooled and sequenced on an Illumina NovaSeq 6000 sequencer according to the effective library concentration and data amount in 150 ×150 paired-end mode. After sequencing, the base-called demultiplexed (fastq) read qualities were determined using FastQC (v0.11.2)^32^ and aligned to the GENCODE v32 human genome (GRCh38.v32) from human samples and the GENCODE M23 mouse genome (GRCm38. M23) for mouse samples and gene counts generated using STAR (v2.7.3a).^33^ Datasets are availble at the NCBI GEO repository with series accession number GSE250357. Postalignment qualities were generated with Picard tools. An expression matrix of the raw gene counts was generated using R^34^ and filtered to remove genes with low counts (defined as those with fewer than 5 reads in at least one sample). The filtered expression matrix was used to generate a list of differentially expressed genes between the sample groups using DESeq2,^35^ and the genes were analyzed via principal component analysis (PCA). Reads corresponding to chromosomal regions chr20:909,365 and chr20:58,909,366 were analyzed to detect and quantify the GNAS p.R201C (C > T) and pR201H (G > A) substitutions, respectively. An FD tissue genetic signature comprising 276 genes was derived by comparing WT versus Gnas R201C-expressing mice; the genes had a > 2 log_2_-fold change and were significantly (adjusted *P* value ≤ 0.05) differentially expressed between the two groups. This gene signature was converted to human genes based on homology scores using the Biomart service, which reduced the number of genes to 202. Similarly, a list of genes positively correlated with the proliferation marker PCNA in healthy human tissues was compiled from Venet et al.^10^ A custom.gmt file was compiled from the FD, meta-PCNA signature and MSigDB GO-derived gene sets corresponding to osteoclast and osteoblast differentiation and activity biological processes. For osteoblasts, the gene set GO:0001649 “osteoblast differentiation” list was used. For osteoclasts, we combined two gene sets: GO:0030316 “osteoclast differentiation” and GO:0045453 “bone resorption”. The gene set variation analysis (GSVA)^36^ method was used to compute enrichment scores for each sample against these selected gene sets using the Poisson kernel, and the resulting matrix was subjected to unsupervised clustering using the Euclidean metric. Selected gene expression heatmaps were generated from log-transformed TMM-normalized gene counts using a heatmap.2R function.

### Ex vivo FD cultures

Bone marrow explants were isolated and cultured as described previously.^11^ Briefly, the tibiae and femurs were dissected from uninduced mice or wild-type littermates, the marrow cavities were opened by drilling small holes in the epiphyses using hypodermic needles, and the marrow was flushed using a syringe containing culture media. The isolated marrow was then triturated and added to a T-75 culture flask supplemented with complete explant media (α-MEM supplemented with 20% fetal bovine serum and 1x penicillin/streptomycin) supplemented with 1x Normocin (ant-nr-1; InvivoGen) during culture establishment. The cells were left undisturbed in a cell culture incubator for 10 days, after which a mixed culture of predominantly BMSCs and osteoclast precursors was established. Cultures were subsequently passaged via 0.05% trypsin washing, lifted via cell scrapers and used for downstream experiments. Cultures below passage 4 were used for the experiments. For FD induction, cells were plated at ~40% confluency in 6-well plates, 24-well plates, or angiogenesis µ-slides (Cat No. 81506 Ibidi, Firchburg, WI) and treated with 5 µM doxycycline (Sigma, # D9891-5G) to activate Gα_s_^R201C^-expressing BMSC lineage cells. During induction, the media were refreshed daily. Initial osteoclast fusion was typically observed after 3 days of induction, and samples were collected after 4 days of induction. Neutralizing RANKL antibody (BE0191, Bioxcell) or IgG2a (BE0089, Bioxcell) was added at 1 µg/mL at the beginning of induction, after which the cells were maintained throughout the experiment. MC3T3-E1 clones 4 and 14 were cultured as previously described.^31^

The human BMSCs cultured for BaseScope optimization were cultured as previously described.^15^

### Histology and cell culture staining

#### Tissue and culture preparation

Dissected mouse hindlimbs and human baseline and post-treatment bone biopsies were fixed in Z-fix (Anatech, USA) overnight at 4 °C. Samples for paraffin embedding (PE) were decalcified in 0.25 mol/L EDTA at 4 °C. The samples were then embedded and sectioned into 5 µm sections. The slides were stored at 4 °C or –20 °C until staining. Sections from all PE samples were deparaffinized in xylene and rehydrated using a graded ethanol series for subsequent staining. Tissue morphology was assessed by staining sections with H&E.

Tissue samples for cryosectioning were shipped to Bonebase (at UConn Health, Farmington, CT) in formalin and in wet ice and embedded and cryosectioned following the methods of Dyment et al.^37^

The cell cultures were fixed with warm, freshly prepared 4% formaldehyde in PBS (Sigma, F1268) at 37 °C.

Patient and healthy volunteer-derived BMSC cultures were pelleted at 4 °C and 300 × *g* for 5 min, embedded in OCT media, frozen and cryosectioned for Gα_s_ mRNA detection, optimization and validation.

#### TRAP and ALP enzymatic detection

Mouse cryosections were stained for TRAP and ALP enzymatic activity at Bonebase following previously described methods.^37^ Mouse culture TRAP staining was performed with a Cosmo Bio TRAP Staining Kit (Cat No. PMC-AK04F-COS, Cosmo, Carlsbad, CA). Human PE sections were incubated with TRAP staining solution (Wako, Cat No. 294-67001) for 25 min at RT, counterstained with Methyl Green and mounted using EcoMount.

#### Immunohistochemistry

PE sections were deparaffinized in xylene and rehydrated using a graded ethanol series. Endogenous peroxidase activity was blocked using 3% H_2_O_2_ in methanol. For Runx2 and MCM2, antigen retrieval was performed using a Uni-Trieve (Innovex Biosciences, Cat No. NB325) for 30 min at 55 °C or 45 min at 70 °C, respectively. Nonspecific binding was blocked using goat or rabbit serum (Vector, Cat No. PK-6105 and PK-6101) as appropriate. Next, both human and mouse sections were incubated with rabbit anti-Runx2 (1:400; Abcam, Cat No. ab192259), or rabbit anti-Mcm2 (1:200; Abcam, Cat No. 108935); human sections were incubated with rabbit anti-osteocalcin (1:200; Proteintech, Cat No. 23418-1-AP); and mouse sections were incubated with goat anti-sclerostin (1:100; R&D Systems, AF1589) overnight at 4 °C. Rabbit and goat isotype control antibodies were used at similar concentrations (BioLegend, Cat No. 910801 and R&D Systems, Cat No. AB-108-C, respectively), and no significant nonspecific staining was observed (not shown). The sections were then incubated with the corresponding goat anti-rabbit or rabbit anti-goat biotinylated secondary antibodies (Vector, Cat No. PK-6105 and PK-6101) for 45 min at RT, followed by incubation with VECTASTAIN Elite ABC Reagent (Vector, Cat No. PK-6100) for 30 min at RT. Finally, the sections were stained with DAB-EASY tablets (Acros Organics, Thermo Fisher Scientific, Cat No. AC328005000) until the desired staining intensity was achieved. Samples were counterstained with Methyl Green (Vector, H-3402-500) and mounted using EcoMount (Biocare Medical, Cat No. EM897L).

#### In vitro fluorescence and immunofluorescence

Mouse marrow explant cultures were stained with antibodies against Tsg101 (NOVUS, 4A10), Runx2 (Abcam, 76956), Ki-67 (CST, D3B5) and Rank (Abcam, 13918). Mouse Alexa Fluor 555 (CST, 4409) and rabbit Alexa Fluor 647 (CST, 4414) were used as fluorescent secondary antibodies. Osteoclast fusion was evaluated via fluorescence microscopy as previously described^38^ using phalloidin-Alexa488 and Hoechst (Invitrogen, Cat Nos. H3570 and A30106, respectively) to label the actin cytoskeleton and nuclei. For all the stains, the cells were permeabilized for 10 min in PBS supplemented with 0.1% Triton X-100, and 5% FBS (IF Buffer) was used to suppress nonspecific binding. Then, the cells were incubated with primary antibodies overnight in IF buffer. After washing in IF buffer, the cells were incubated for 2 h at room temperature in IF buffer with secondary antibodies. Images were captured on a Zeiss LSM 800 Airyscan confocal microscope using a C-Apochromat 63x/1.2 water immersion objective.

#### In situ hybridization

For human *SOST* mRNA hybridization, sections were deparaffinized and incubated with RNAscope Hydrogen Peroxide (Advanced Cell Diagnostics [ACD], Cat No. 322335) for 10 min at RT. Target retrieval was performed using ACD Custom Pretreatment Reagent (ACD, Cat No. 300040) for 45 min at 40 °C. Probes were hybridized as instructed and consisted of *dapB* (bacterial gene, negative control), *PPIB* (housekeeping gene), and *SOST* (sclerostin). The remaining hybridization steps were performed using RNAscope 2.5 HD Detection Reagents- RED (ACD, Cat No. 322360) with the exception of AMP5, which was incubated for 60 min. Sections were counterstained with 50% Gil’s Hematoxylin I (Sigma‒Aldrich, Cat No. GHS132) and mounted with EcoMount (Biocare Medical, Cat No. EM897L).

In situ hybridization of Gα_s_ p.R201 variant mRNAs was performed using the novel Advanced Cell Diagnostics [ACD] Basescope duplex system with custom-made probes following the manufacturer’s protocols. For optimization and validation of the method, human bone marrow stromal cell (BMSC) primary cultures containing GNAS p.R201C, GNAS pR201H and WT variants were cultured, pelleted by centrifugation and embedded in OCT cryosectioning media. Five-micron sections were assessed for RNA hybridization of two candidate probes for each GNAS p.R201C, GNAS pR201H and WT variant in collaboration with the ACD team. Images of hybridization with the probes validated for further studies are shown in Fig. S4a.

Briefly, human sections were incubated with RNAscope Hydrogen Peroxide (ACD, Cat No. 322335) for 10 min at RT and retrieved with ACD Custom Pretreatment Reagent (ACD, Cat No. 300040) for 60 min at 40 °C. The positive controls used were *PPIB* (high-expression housekeeping gene) and *POLR2A* (low-expression housekeeping gene), and the negative controls used were *dapB* (bacterial gene) probes and custom-designed probes for wild-type *GNAS* (ACD, Cat No. 1061061-C1), as well as the two most common mutations in FD: GNASR201C (c.601 C > T, Cat No. 1061041-C2) and GNASR201H (c.602 G > A, ACD, Cat No. 1061051-C2). With the BaseScope Duplex Detection Reagent Kit (ACD, Cat No. 323810), the AMP7 and AMP11 concentrations were increased at 45 min and 60 min, respectively, to increase the staining intensity. Sections were counterstained using 50% Gil’s hematoxylin I and mounted with VectaMount Permanent Mounting Medium (Vector, Cat No. H-5000). The results of staining with the positive and negative control probes are shown in Fig. S4b-g.

#### Microscopy imaging

Chromogenically stained PE sections were scanned using a NanoZoomer S60 Digital slide scanner (Hamamatsu, Cat No. C13210-01) at 400 × magnification.

Fluorescent cryogenic sections were scanned using an Axioscan 7 fluorescence scanner (Zeiss, Oberkochen, Germany)^37^ with appropriate filters for the detection of DAPI (Em 460 nm, Ab 350 nm), Elf97 TRAP (Em 550 nm, Ab 375 nm) and ALP (Em 605 nm, Ab 545 nm). The scanned slides were manually aligned and converted to Adobe Photoshop.psd files.

Cell immunofluorescence images were captured on a Zeiss LSM 800 Airyscan confocal microscope using a C-Apochromat 63x/1.2 water immersion objective. For the osteoclast fusion assay, 8 randomly selected fields of view were imaged using Alexa 488, Hoechst and phase contrast compatible filter sets (BioTek) on a Lionheart FX microscope using a 10x/0.3 NA Plan Fluorite WD objective lens (BioTek) with Gen3.10 software (BioTek).

#### Microscopy quantification

For bone content and cellularity, images from H&E-stained PE sections were analyzed using a custom script in ImageJ (NIH). For human sections, complete biopsy sections were analyzed. For mice, 1–3 fields of 0.6 by 0.4 mm of randomly selected FD lesion tissue were analyzed per sample. Nonbone or non-FD tissue was removed using Adobe Photoshop 2021 when necessary (Adobe, San Jose, CA). On ImageJ, pixel-to-area calibration was performed, mineralized and fibrous tissue areas were manually traced, and nuclei were selected in these areas using a semiautomatic color threshold selection followed by a watershed separation plugin. The number of nuclei and the mineralized and fibrotic areas were calculated using the “analyze particles” function. The total number of nuclei obtained with this method was used as the denominator to calculate the ratio of cells positive for TRAP activity, Rank, and Mcm2 in consecutive sections from the human biopsies.

For mouse TRAP and ALP analysis of mouse FD cryosections, multiplexed fluorescence 1 mm × 1 mm images of the distal tibiae were opened in Adobe Photoshop, and the color levels were adjusted and exported as single-layer tiff files corresponding to TRAP, ALP or DAPI for image quantification. The images were subsequently analyzed with QuPath version 0.3.2, an open-source software for digital pathology analysis.^39^ After calibration of the pixel-to-area ratio of the images, cell nuclei were identified by DAPI staining and counted using the “cell detection” function. TRAP- and ALP-stained areas were traced using the “pixel classification” function, in which the area of fluorescence (µm^2^) was calculated based on a pixel color intensity threshold. The average TRAP and ALP staining intensity per cell was calculated as the ratio of the µm^2^ TRAP- or ALP-positive area to the number of nuclei in each quantified image.

We used the QuPath tool in PE scans to manually label and count cells positive for TRAP, Rank (multinuclear osteoclasts or mononuclear precursors), Mcm2 and sclerostin-stained (either by RNA hybridization in humans or immunohistochemistry in mice) and unstained osteocytes. For ACD Basescope™ quantification, we used this tool on 400x images to label hematoxylin-stained nuclei in proximity to the detected Gα_s_^R201C^ or Gα_s_^R201H^ mRNA molecules (red dots), quantified as mutant cells, regardless of the presence of neighboring wild-type Gα_s_ mRNA molecules (green dots). Nuclei associated with only wild-type Gα_s_ mRNA molecules were quantified as wild-type cells. The positive controls *PPIB* (a high-expression housekeeping gene) and *POLR2A* (a low-expression housekeeping gene) and the negative control *dapB* (a characteristic bacterial gene) were used.

Osteoclast fusion efficiency was evaluated by the number of fusion events between osteoclasts with obvious ruffled borders and ≥3 nuclei in phalloidin-Alexa 488/Hoechst images, as described previously.^40^ Since, regardless of the sequence of fusion events, the number of cell-to-cell fusion events required to generate a syncytium with N nuclei is always equal to N-1, we calculated the fusion number index as Σ (Ni − 1) = Ntotal − Nsyn, where Ni = the number of nuclei in individual syncytia and Nsyn = the total number of syncytia.

RUNX2 and OCN staining in human samples was quantified by a scoring method since the variable intensity of staining made it impossible to quantify based on a cell positivity color threshold. For the secreted factor OCN, the stain was normally dispersed and not associated with individual cells; therefore, a collection of 100x-blinded microscopy images was studied and assigned to 5 scores based on their staining level (0, no stain; 5, maximum stain). Two images of each staining level were subsequently provided to the readers for training purposes. For RUNX2, descriptive scores were developed and are shown in Table S2. All assessments that required human evaluation were examined by at least 3 trained readers in a blinded fashion.

### In vitro bone resorption assay

Bone resorption was evaluated using a bone resorption assay kit from Cosmo Bio (USA) according to the manufacturer’s instructions. In brief, fluoresceinamine-labeled chondroitin sulfate was used to label 24-well, calcium phosphate-coated plates. Media were collected at 4-5 days post-induction, and the fluorescence of the media was evaluated as recommended by the manufacturer.

### Cultured cell cytometry analyses

BrdU incorporation was used to evaluate the fraction of cells undergoing DNA replication as a surrogate for cell proliferation using a FITC BrdU Flow Kit (BD Pharmingen) according to the manufacturer’s instructions, with one exception. To avoid interference due to GFP expression in combination with Gα_s_^R201C^, we utilized an α-BrdU-Alexa 555 antibody. Briefly, BrdU was added to media for 4 hours in uninduced cultures, and cultures induced with doxycycline for 4 days that were treated with αRANKL or an isotype control antibody. We also induced cultures that were not labeled with BrdU to act as controls for both autofluorescence and nonspecific antibody labeling. The cells were subsequently digested with trypsin, fixed, and cryogenically preserved until staining and analysis. Approximately 5 000 gated cells were evaluated for BrdU incorporation per condition per replicate. The cells were analyzed using a NovaCyte flow cytometer (ACEA Biosciences, Inc.) with NovoExpress software (v1.5.0) at a rate of <150 events/second.

### Culture media determinations

Ex vivo cultures were cultured in complete culture media supplemented with FBS depleted of EVs (via ultracentrifugation at 150 000 × *g* for >2 hours). Twenty-four hours later, the conditioned media was collected, and the cells/large cell debris were depleted via centrifugation (15 min at 4 000 × *g*).

#### Enrichment and quantification of extracellular vesicle (EV) fractions

The EV fraction was enriched via ultracentrifugation (150 000 x *g* for 1.5 hours). Alternatively, an exoEasy Maxi Kit (Qiagen, Hilden Germany) was used to isolate EVs. EV-enriched fractions were evaluated via Western blotting using anti-Tsg101 (Cat No. 4A10; NOVUS, Louis, MO) and anti-RANK (Cat No. 13918; Abcam, Cambridge, UK) antibodies. Tsg101 and Rank band staining were quantified via densitometry via ImageJ.

#### Quantification of released factors in culture media

The levels of RANKL, M-CSF, SEMA3A, EFNB2 and FAS-L (undetectable) were determined in culture media with the following ELISA kits per the manufacturer’s instructions: MTR00 Mouse TRANCE/RANKL/TNFSF11 Quantikine ELISA Kit, MMC00B Mouse M-CSF Quantikine ELISA Kit, MFL00 Mouse Fas Ligand/TNFSF6 Quantikine ELISA Kit (R&D Systems), LS-F33608 Mouse SEMA3A ELISA Kit, and LS-F6974 Mouse EFNB2 ELISA Kit (LSBio LifeSpan Biosciences, Inc.).

### Statistical analyses

The data are expressed as individual data points and means ± SEMs for all values. The results were tested for normality using the Shapiro‒Wilk test, and analyses were performed using nonpaired t tests or Mann‒Whitney tests. For longitudinal measures, paired two-tailed t tests were used to compare baseline values to values at different time points. The data are reported as the average and standard deviation when shown, unless otherwise indicated, and analyses were conducted on GraphPad Prism 8.0.2 by LFdC. Culture data were evaluated by two-tailed t tests paired with donor mouse data and paired t tests for MC3T3-E1 experimental data from JW.

### Study approval

The clinical trial NCT03571191 was approved by the NIH Investigational Review Board, and informed consent was obtained from all the subjects. The study was monitored by a data safety and monitoring committee organized by the National Institute of Dental and Craniofacial Research. Mouse experiments were conducted under a protocol approved by the NIH/NIDCR Animal Care and Use Committee (ASP 19-897).

### Supplementary information

Suplementary figures 1-6

Supplementary tables S1-S2

Supplementary tables S3-S8
