# Supplementary material for: RANKL inhibition reduces lesional cellularity and Gαs variant expression and enables osteogenic maturation in fibrous dysplasia
Source: Bone Res. 2024 Feb 20;12:10. doi: 10.1038/s41413-023-00311-7 (PMC10879491; doi:10.1038/s41413-023-00311-7)
Supplement: Supplementary file 1 — Suplementary figures 1-6 [file 41413_2023_311_MOESM1_ESM.pdf]

Fig S1

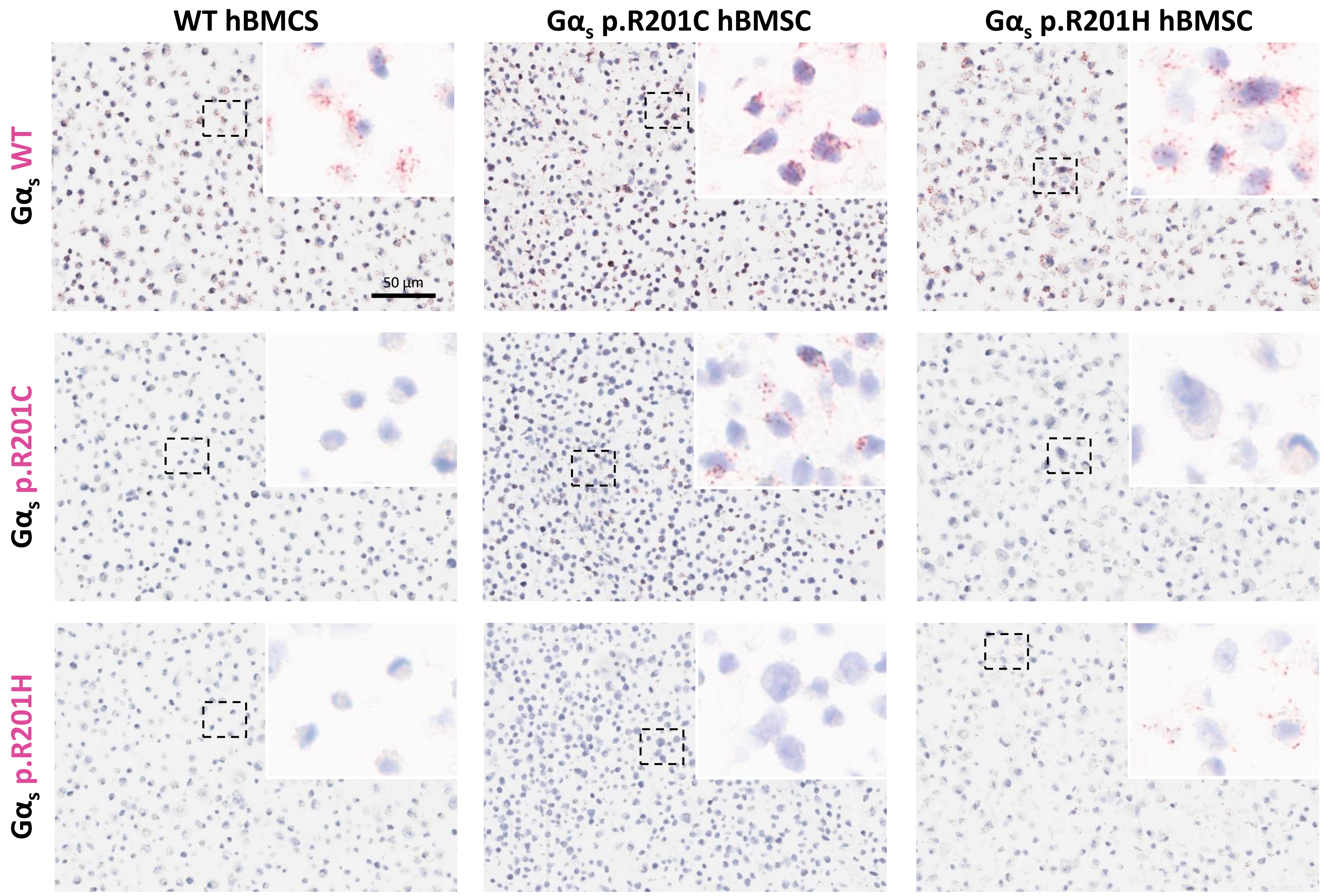
WT hBMCS
Gα$_s$ p.R201C hBMSC
Gα$_s$ p.R201H hBMSC
Gα$_s$ WT
Gα$_s$ p.R201C
Gα$_s$ p.R201H
50 µm

Fig S2

A

FD Patients

Subject ID	Age (years) & sex	MAS endocrinopathies	Biopsy location
DB02	54F	Precocious puberty	Rib
DB04	26F	GH excess	Rib
DB05	34F	Precocious puberty	Rib
DB07	40F	Precocious puberty	Scapula
DB08	30F	Precocious puberty	Iliac crest
DB09	20F	Precocious puberty	Rib

Patient treatment regimen

- Baseline biopsy #1
- Denosumab 120 mg every 4 weeks, with loading doses on weeks 2 & 3
- 6-month biopsy #2

FD Mouse Model

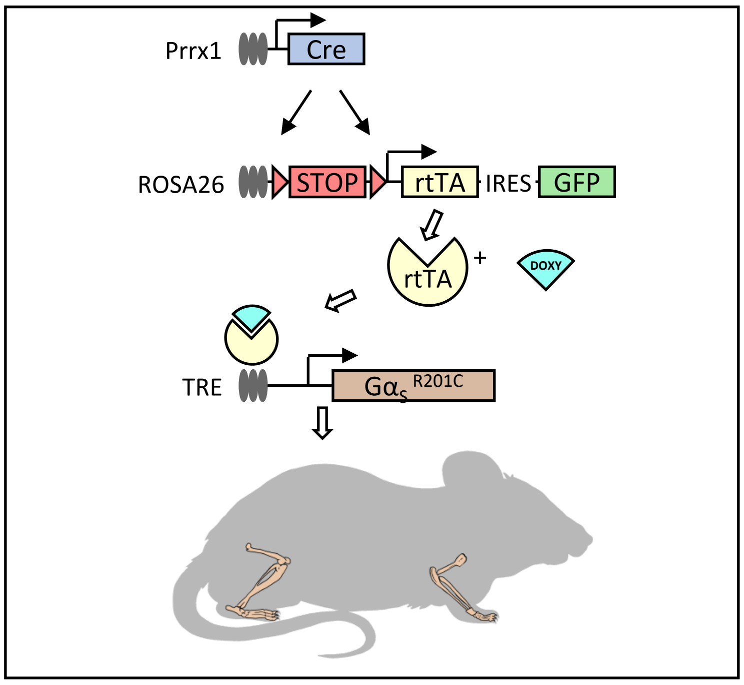

FD Induction and treatment

- FD lesions induced for 28 days
- αRANKL or IgG (6 mg/Kg) on days 28, 30, 35, 42, 49 and 56
- Euthanized on day 58

B

Technique in patients	**(Analysis)**	Technique in mice
H&E, MCM2 (fig. 2)	**(Lesion cellularity and proliferation)**	In vivo: H&E, MCM2 (fig. 4)<br>In vitro: Ki67+Runx2 IF, BrdU FACS (fig. 7)
RANK, TRAP (fig, 2)	**(Lesion osteoclastic differentiation and activity)**	In vivo: TRAP (fig. 4)<br>In vitro: Fusion assay, RANK+ Evs; RANKL, M-CSF, SEMA3A, EPHB4, EFNB2 and FAS-L release in media (fig. 6)
Basescope $G\alpha_s$ variant detection, mRNAseq variant detection (fig. 1, S1)	**(Mutation burden)**	(not applicable)
RUNX2, OC, SOST (fig. 2)	**(Lesion osteogenic differentiation)**	RUNX2, ALP, Sost (fig. 4)
Site-matched biopsies (fig. 5, S5)	**(mRNA seq)**	WT, FD+IgG (control), FD+αRANKL (fig 5, S5)

Fig S3

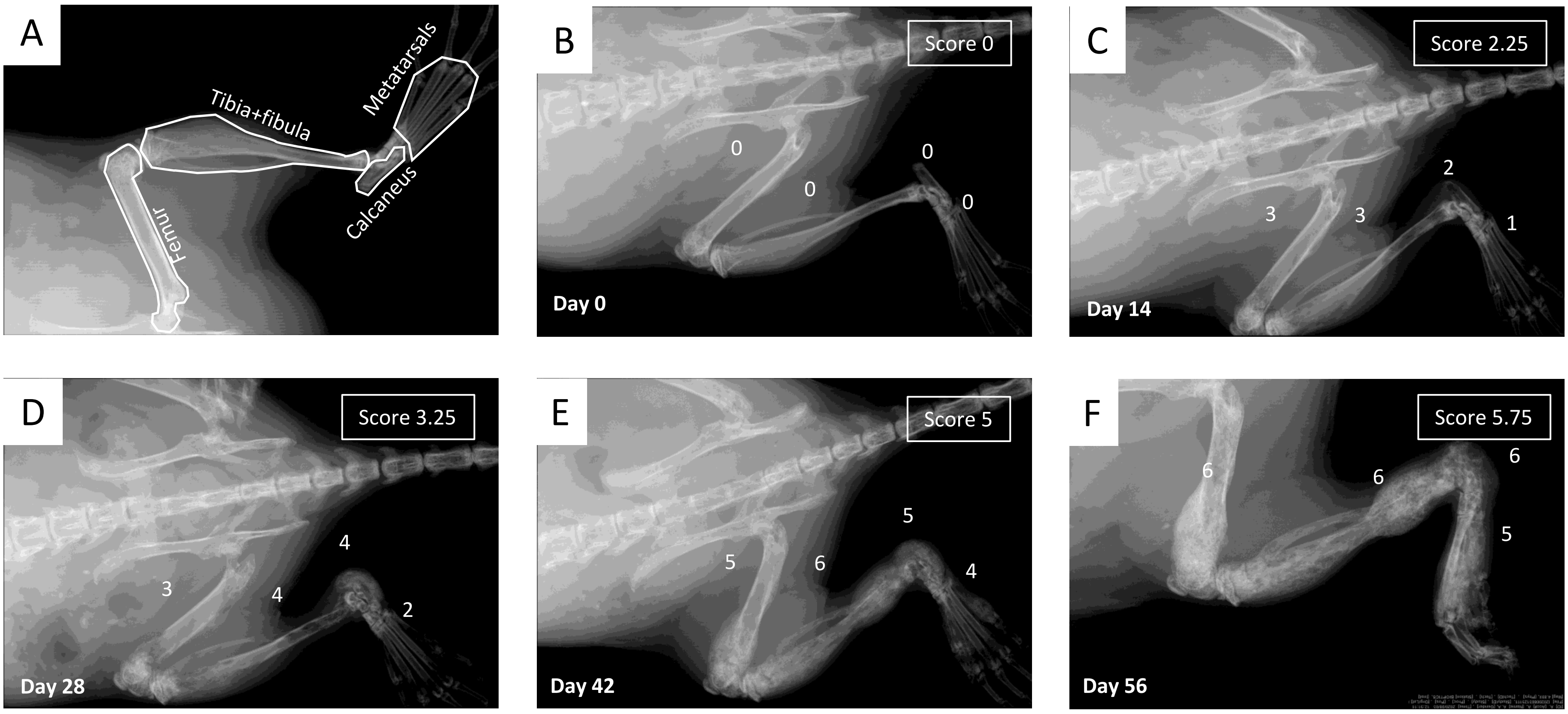

A
Metatarsals
Tibia+fibula
Calcaneus
Femur
B
Score 0
0
0
0
0
Day 0
C
Score 2.25
2
3
3
1
Day 14
D
Score 3.25
4
3
4
2
Day 28
E
Score 5
5
5
6
4
Day 42
F
Score 5.75
6
6
6
5
Day 56

Fig S4

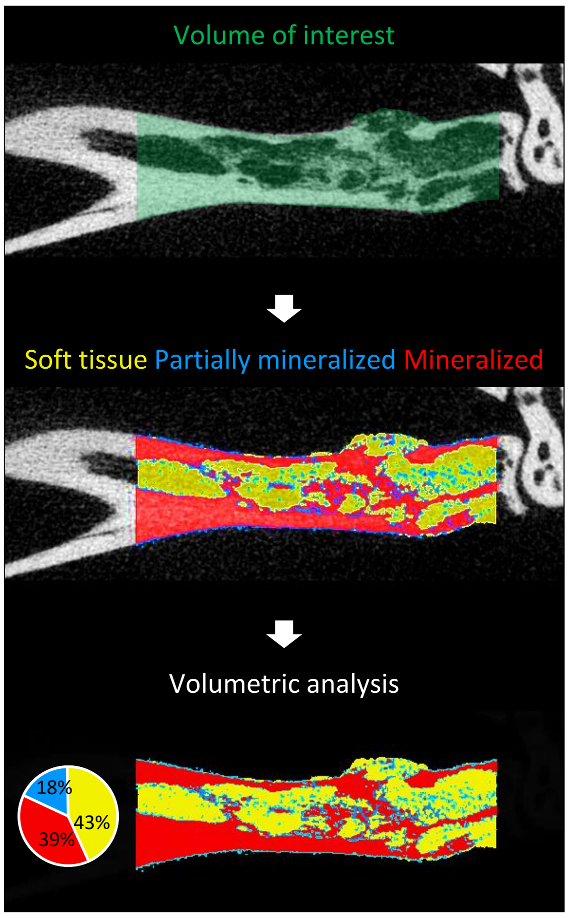
Volume of interest
Soft tissue Partially mineralized Mineralized
Volumetric analysis
18%
43%
39%

Fig S5

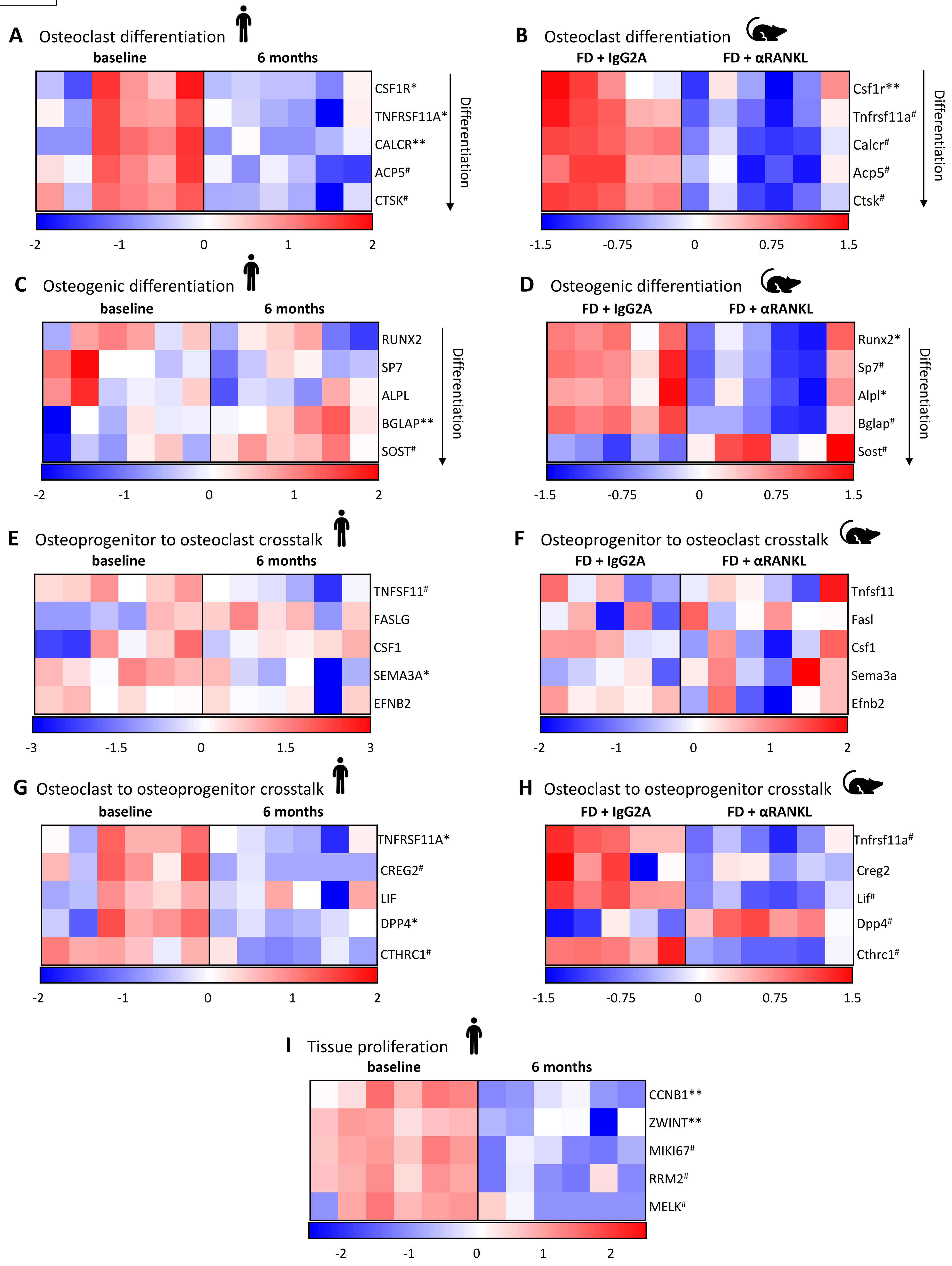
A Osteoclast differentiation
baseline
6 months
CSF1R*
TNFRSF11A*
CALCR**
ACP5#
CTSK#
Differentiation
-2
-1
0
1
2
B Osteoclast differentiation
FD + IgG2A
FD + αRANKL
Csf1r**
Tnfrsf11a#
Calcr#
Acp5#
Ctsk#
Differentiation
-1.5
-0.75
0
0.75
1.5
C Osteogenic differentiation
baseline
6 months
RUNX2
SP7
ALPL
BGLAP**
SOST#
Differentiation
D Osteogenic differentiation
FD + IgG2A
FD + αRANKL
Runx2*
Sp7#
Alpl*
Bglap#
Sost#
Differentiation
E Osteoprogenitor to osteoclast crosstalk
baseline
6 months
TNFSF11#
FASLG
CSF1
SEMA3A*
EFNB2
-3
-1.5
0
1.5
3
F Osteoprogenitor to osteoclast crosstalk
FD + IgG2A
FD + αRANKL
Tnfsf11
Fasl
Csf1
Sema3a
Efnb2
G Osteoclast to osteoprogenitor crosstalk
baseline
6 months
TNFRSF11A*
CREG2#
LIF
DPP4*
CTHRC1#
H Osteoclast to osteoprogenitor crosstalk
FD + IgG2A
FD + αRANKL
Tnfrsf11a#
Creg2
Lif#
Dpp4#
Cthrc1#
I Tissue proliferation
baseline
6 months
CCNB1**
ZWINT**
MIKI67#
RRM2#
MELK#

Fig S6

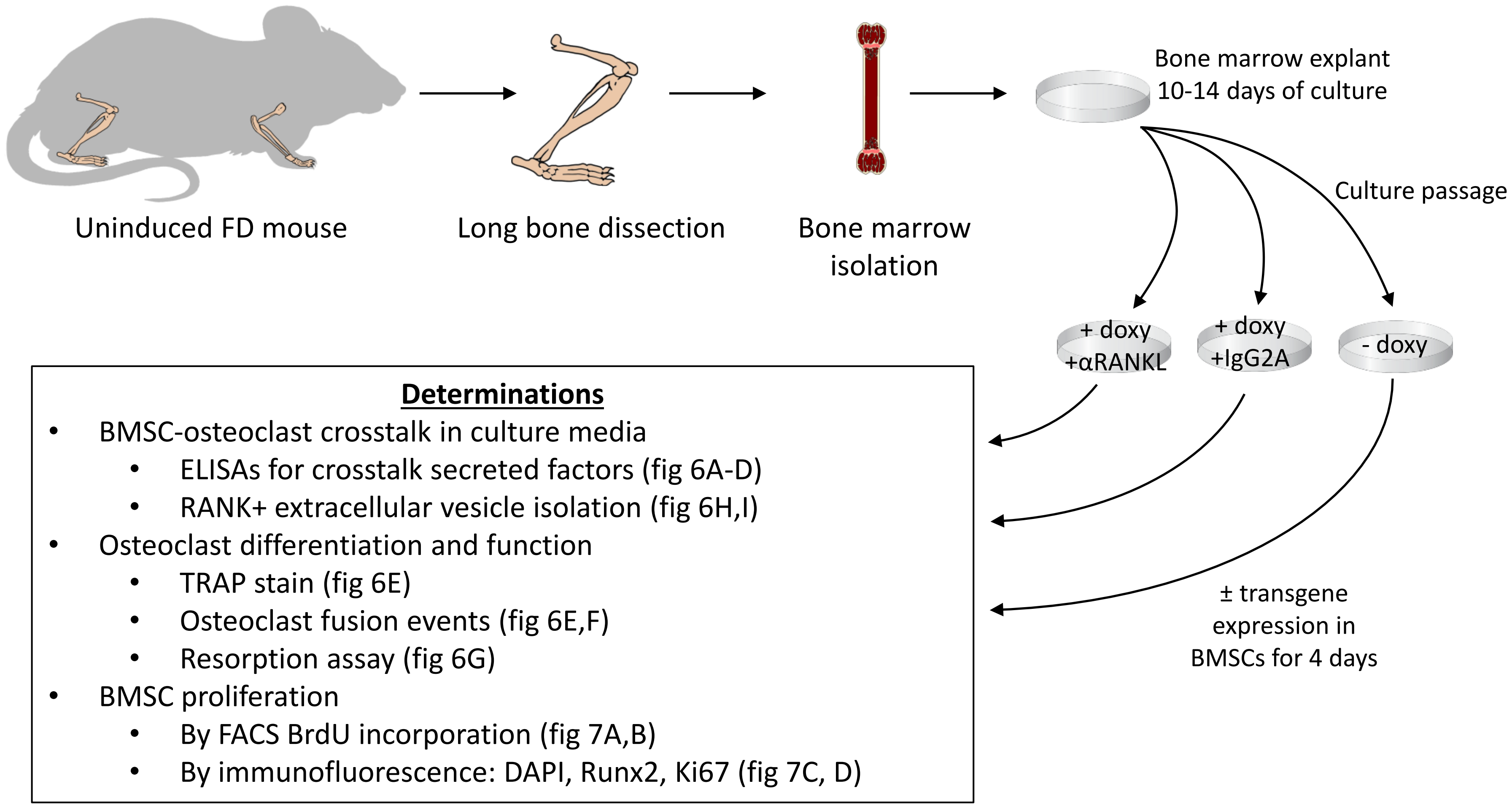

Uninduced FD mouse
Long bone dissection
Bone marrow isolation
Bone marrow explant 10-14 days of culture
Culture passage
+ doxy +αRANKL
+ doxy +IgG2A
- doxy
± transgene expression in BMSCs for 4 days
Determinations
• BMSC-osteoclast crosstalk in culture media
• ELISAs for crosstalk secreted factors (fig 6A-D)
• RANK+ extracellular vesicle isolation (fig 6H,I)
• Osteoclast differentiation and function
• TRAP stain (fig 6E)
• Osteoclast fusion events (fig 6E,F)
• Resorption assay (fig 6G)
• BMSC proliferation
• By FACS BrdU incorporation (fig 7A,B)
• By immunofluorescence: DAPI, Runx2, Ki67 (fig 7C, D)
