# Supplementary material for: RANKL inhibition reduces lesional cellularity and Gαs variant expression and enables osteogenic maturation in fibrous dysplasia
Source: Bone Res. 2024 Feb 20;12:10. doi: 10.1038/s41413-023-00311-7 (PMC10879491; doi:10.1038/s41413-023-00311-7)
Supplement: Supplementary file 2 — Supplementary tables S1-S2 [file 41413_2023_311_MOESM2_ESM.docx]

## Table S1: Mouse X-ray disease burden score

**Mouse disease burden score = (left femur + left tibia-fibula + left calcaneus + left metatarsals + right femur + right tibia-fibula + right calcaneus + right metatarsals) / 8**

| **Score 🡪**  **Bone(s) ↓** | **1** | **2** | **3** | **4** | **5** | **6** |
| --- | --- | --- | --- | --- | --- | --- |
| **Femur** | Focal lytic areas covering <10% of the femur Typically just one spot in the distal metaphysis. | Extended lytic areas covering 10-25% of the femur.  OR Focal sclerotic only covering <10% of the femur and immediately proximal to the distal metaphysis | Lytic or lytic-sclerotic changes, covering 25-50% of the femur No Expansion | Lytic or lytic-sclerotic changes, covering >50% of the femur No Expansion | Lytic or lytic-sclerotic changes, covering >50% of the femur Mild Expansion, diaphysis width <125% control | Lytic or lytic-sclerotic changes, covering >50% of the femur Severe Expansion, diaphysis width >125% control |
| **Tibia-fibula** | Focal lytic areas covering <10% of the tibia Typically just one spot in the distal submetaphysis. Rarely more proximal in the diaphysis or in the tibial crest | Extended lytic areas covering 10-25% of the tibia No Expansion Mid-diaphysis unaffected | Lytic/sclerotic areas covering 25-50% of the tibia No Expansion Mid-diaphysis unaffected Typically no lesioned tissue proximal to the fibula insertion | Lytic/sclerotic areas covering 50-75% of the tibia with mild bowing (score 4a) OR lytic/sclerotic areas covering 25-50% of the tibia with focal expansion <150% in size (score 4b) | Lytic/sclerotic changes, covering 50-75% % of the tibia Focal Expansion - width >150% in size | Lytic/sclerotic changes, covering over 75% of the tibia Spread Expansion - width >150% normal |
| **Calcaneus** | Focal lytic areas No expansion | Extended lytic or lytic/sclerotic areas No expansion | Expansion Size 100-125% normal Lytic/sclerotic | Expansion Size >125% normal No ankylosis of the ankle Lytic/sclerotic | Expansion Size >125% normal Partial ankylosis of the ankle Lytic/sclerotic | Expansion Size >125% normal Complete ankylosis of the ankle Lytic/sclerotic |
| **Metatarsals** | Focal lytic areas in some metatarsals, immediately proximal to the distal metaphysis. It can be differentiated from a low-capture X-ray because in the radiolucent cortex is restricted to these areas (not continuous) | Extended lytic areas in some of the metatarsals. No Expansion | Expansion Width of affected bones < 150% Lytic and sclerotic | Expansion Width of affected bones 150-200% Unaffected areas in at least 1 metatarsal Lytic and sclerotic | Expansion Width of affected bones >200% Unaffected areas in at least 1 metatarsal Lytic and sclerotic | Expansion Width of affected bones >200% No unaffected areas Lytic and sclerotic |

Table S2: Scoring method for human RUNX2 immunohistochemistry

| **Score** | **Runx staining** |
| --- | --- |
| 0 | no stain |
| 1 | Less than 10% cells stained |
| 2 | 50% cells dimly stained or up to 25% cells strongly stained |
| 3 | 100% cells dimly stained or 25-50% cells strongly stained |
| 4 | 50-75% of the cells strongly stained, rest dimly stained |
| 5 | 100% cells strongly stained |
